# Distributional records of Antarctic and sub-Antarctic Ophiuroidea from samples curated at the Italian National Antarctic Museum (MNA): check-list update of the group in the Terra Nova Bay area (Ross Sea) and launch of the MNA 3D model ‘virtual gallery’

**DOI:** 10.3897/zookeys.705.13712

**Published:** 2017-10-02

**Authors:** Matteo Cecchetto, Maria Chiara Alvaro, Claudio Ghiglione, Alice Guzzi, Claudio Mazzoli, Paola Piazza, Stefano Schiaparelli

**Affiliations:** 1 Department of Earth, Environmental and Life Science (DISTAV), University of Genoa, Genoa, Italy; 2 Italian National Antarctic Museum (MNA, Section of Genoa), University of Genoa, Genoa, Italy; 3 Department of Geosciences, University of Padua, Padua, Italy; 4 Department of Physical, Earth and Environmental Sciences, University of Siena, Siena, Italy

**Keywords:** Antarctica, Bransfield Strait, Echinodermata, Falkland Islands (Islas Malvinas), MNA, Ophiuroidea, Ross Sea, Terra Nova Bay, virtual collection, Weddell Sea, 3D models

## Abstract

The distributional records of Ophiuroidea stored at the Italian National Antarctic Museum (MNA, Section of Genoa) are presented, corresponding to 1595 individuals that belong to 35 species and 17 genera. Specimens were collected in 106 different sampling stations at depths ranging from 21 to 1652 m in the framework of 14 Antarctic expeditions to the Ross Sea, one to the Antarctic Peninsula, and one to the Falkland Islands (Islas Malvinas). Three species, *Amphiura joubini* Koehler, 1912, Amphiura (Amphiura) angularis Lyman, 1879, and *Ophiura flexibilis* (Koehler, 1911), are reported as new records for the Terra Nova Bay area, whose check-list of species increases from 15 to 18 species. The determination of these three new records was based both on morphological identification and molecular analyses (COI barcoding). Some of the genetically characterised specimens were also documented through photogrammetry and micro-computed tomography and represent the first bulk of 3D models that will be available through the MNA and Sketchfab websites, both for research and educational purposes.

## Introduction

Museum collections have always been indispensable resources for biodiversity studies by providing data over a vast time span (Suarez and Tsutsui 2004). The recent and continuous advance of newly developed molecular techniques and informatics highlighted the importance of (and renewed the interest for) these permanent repositories of specimens, which now may also disclose new information and data, previously not accessible due to technological limitations (Kemp 2015).

In this paper new distributional data is provided for Ophiuroidea collected in the framework of several recent scientific expedition performed in the Southern Ocean and which are now permanently stored and curated at the Italian National Antarctic Museum (MNA, Section of Genoa, Italy). The Ross Sea expeditions were the Italian National Antarctic Program (PNRA) expedition “III” (1987/1988), “V” (1989/1990), “IX” (1993/1994), “X” (1994/1995), “XIII” (1997/1998), “XIV” (1998/1999), “XV” (1999/2000), “XVII” (2001/2002), “XIX” (2003/2004), “XXV” (2009/2010), “XXVII” (2011/2012), “XXVIII” (2012/2013), “XXIX” (2013/2014) and the New Zealand “TAN0802 IPY-CAML Oceans Survey 20/20” voyage (2008). Additional samples collected outside the Ross Sea were obtained from the German ANT-XXIX/3, PS81 expedition (2013) and the US ICEFISH 2004 voyage (2004).

In parallel to this data set a series of ophiuroid 3D models have also been developed based on ‘molecular vouchers’ (i.e. specimens that have also been characterised from a molecular point of view) that are released with the aim of: i) providing the widest accessibility to the MNA collections, ii) helping researchers in crosschecking morphological data of sequenced species from specific geographic areas (in this case species from the Ross Sea and Terra Nova Bay in particular), and iii) producing materials useful for educational purposes. Virtual collections guarantee rapid and simultaneous access to accurate virtual representations of important museum materials, such as type materials (e.g. Faulwetter et al. 2013) or, as in this case, of specimens that have been molecularly characterised, representing a valid support to 2D images and species descriptions. In fact, 3D models enable free exploration through web platforms as well as an ability to print 3D models of fragile Antarctic invertebrates, allowing direct exploration by school students and museum visitors who would never have the opportunity to examine fragile or minute species in detail. Analogously, other researchers from all around the world can have access to these museum materials in a way that catalyse studying opportunities by eliminating the need of shipping precious and sometimes fragile specimens, thus speeding up the process of data sharing. The 3D models here presented are based on micro-computed tomography (Faulwetter et al. 2013; Wipfler et al. 2016) for smaller specimens (i.e. MNA 2644 and MNA 7784) and on photogrammetry for larger ones (i.e. MNA 7368). The distributional dataset is the fourth MNA contribution to the Antarctic Biodiversity Portal, which is the thematic Antarctic node for both the Ocean Biogeographic Information System (AntOBIS) and the Global Biodiversity Information Facility (ANTABIF) (http://www.biodiversity.aq). Previous contributions were: Ghiglione et al. (2013), Piazza et al. (2014) and Selbmann et al. (2015).

## Project description

**Project title**: Antarctic Ophiuroidea in the collection of the Italian National Antarctic Museum (MNA)

**Curator and Promoter**: Stefano Schiaparelli

**Personnel**: Matteo Cecchetto, Maria Chiara Alvaro, Claudio Ghiglione, Alice Guzzi, Claudio Mazzoli, Paola Piazza, Stefano Schiaparelli

**Funding sources**: The Ophiuroidea were collected during different Italian, New Zealand, German and American research projects and expeditions to Antarctica funded by: i) the Italian National Antarctic Research Program (PNRA), ii) the Ministry for Primary Industries (formerly the Ministry of Fisheries, New Zealand Government), iii) the Alfred Wegener Institute Helmholtz Centre for Polar and Marine Research and iv) the National Science Foundation grant to H. William Detrich (North Eastern University). The full list of sponsors is listed below:

**Italian PNRA research projects**:

• 2.1.4.6 (Necton e risorse da pesca) (“III” expedition, 1987/1988)

• 3.2.1.2.5 (Benthos) and 3.2.1.4 (Oceanografia geologica) (“V” expedition, 1989/1990)

• 2d.2 (Ecology and Biogeochemistry of the Southern Ocean) (“IX” expedition, 1993/1994)

• 2d.2 (Ecology and Biogeochemistry of the Southern Ocean) (“X” expedition, 1994/1995)

• 2b.3.1 (Struttura e dinamica delle cenosi marine di Baia Terra Nova) (“XIII” expedition, 1997/1998)

• 2b.3.1 (Struttura e dinamica delle cenosi marine di Baia Terra Nova) (“XIV” expedition, 1998/1999)

• 8.5 (L’area marina protetta di Baia Terra Nova: struttura e variazioni a breve e lungo termine) (“XV” expedition, 1999/2000)

• 4.7 (CARBONANT - Processi genetici e significato paleoclimatico e paleoceanografico dei CARBONati marini biogenici in ANTartide) (“XVII” expedition, 2001/2002)

• 8.5 (L’area marina protetta di Baia Terra Nova: struttura e variazioni a breve e lungo termine) (“XVII” expedition, 2001/2002)

• 1.3 (Ricerche ecofisiologiche ed ecotossicologiche applicate allo studio dei cambiamenti di origine naturale ed antropica che si verificano nell’ambiente antartico) (“XVII” expedition, 2001/2002)

• 2002/8.6 (“The costal ecosystem of Victoria Land coast: distribution and structure along the latitudinal gradient”) (“XIX” expedition, 2003/2004)

• 2006/08.01 (“The coastal ecosystem of Terra Nova Bay” in the Latitudinal Gradient Program - LGP) (“XXV” expedition, 2009/2010)

• 2010/A1.10 (BAMBi; Barcoding of Antarctic Marine Biodiversity) (“XXVII” expedition, 2011/2012)

• 2009/A1.09 (Diversità genetica spazio temporale di endoparassiti delle regioni polari: uno studio per la valutazione dell’impatto dei cambiamenti globali sulle reti trofiche marine) (“XXVIII” expedition, 2012/2013)

• 2010/A1.10 (BAMBi; Barcoding of Antarctic Marine Biodiversity) (“XXVIII” expedition, 2012/2013)

• 2010/A1.11 (Vulnerabilità dei pesci polari al cambiamento climatico: ciclo vitale, habitats e relazione con il ghiaccio marino in *Pleuragramma antarcticum*) (“XXVIII” expedition, 2012/2013)

• 2013/AZ1.15 (ISOBIOTOX: ISOtopi stabili e marcatori molecolari per la ricostruzione di reti trofiche antartiche soggette alla dinamica dei ghiacci marini) (“XXIX” expedition, 2013/2014)

• 2013/AZ1.18 (RAISE: Ricerche integrate sulla ecologia dell’Antarctic Silverfish nel MarE di Ross) (“XXIX” expedition, 2013/2014)

**New Zealand research projects**:

• IPY-CAML voyage (TAN0802, 2008, R/V “Tangaroa”) - Census of Antarctic Marine Life programme - funded by the Government of New Zealand and administered by the Ocean Survey 20/20 CAML Advisor Group (Land Information New Zealand and the Ministry of Fisheries, Antarctica New Zealand, Ministry of Foreign Affairs and Trade and NIWA)

**German research projects**:

• Project 3.1 (Macrobenthic community analysis and biodiversity study) (ANT-XXIX/3 PS81 expedition, 2013, R/V “Polarstern”) (M.C. Alvaro *legit*)

**American research project**:

• ICEFISH 2004 Cruise expedition, the first comprehensive international survey of the fishes of the Sub-Antarctic marine habitat (http://www.icefish.neu.edu) (M. Vacchi *legit*)

**Study area description**: Sampling took place along the Victoria Land in the Ross Sea, in the Bransfield Strait (Antarctic Peninsula) and southeast Falkland Islands (Islas Malvinas). The bathymetric range of sampling stations was from 21 to 1652 m.

**Design description**: Data were assembled by revising Ophiuroidea voucher specimens curated by the Italian National Antarctic Museum collection (MNA), Section of Genoa, Genoa (Italy). These samples were collected in the framework of the above reported research expeditions, which had different aims and geographical scopes.

## Methods

**Figure 1.**
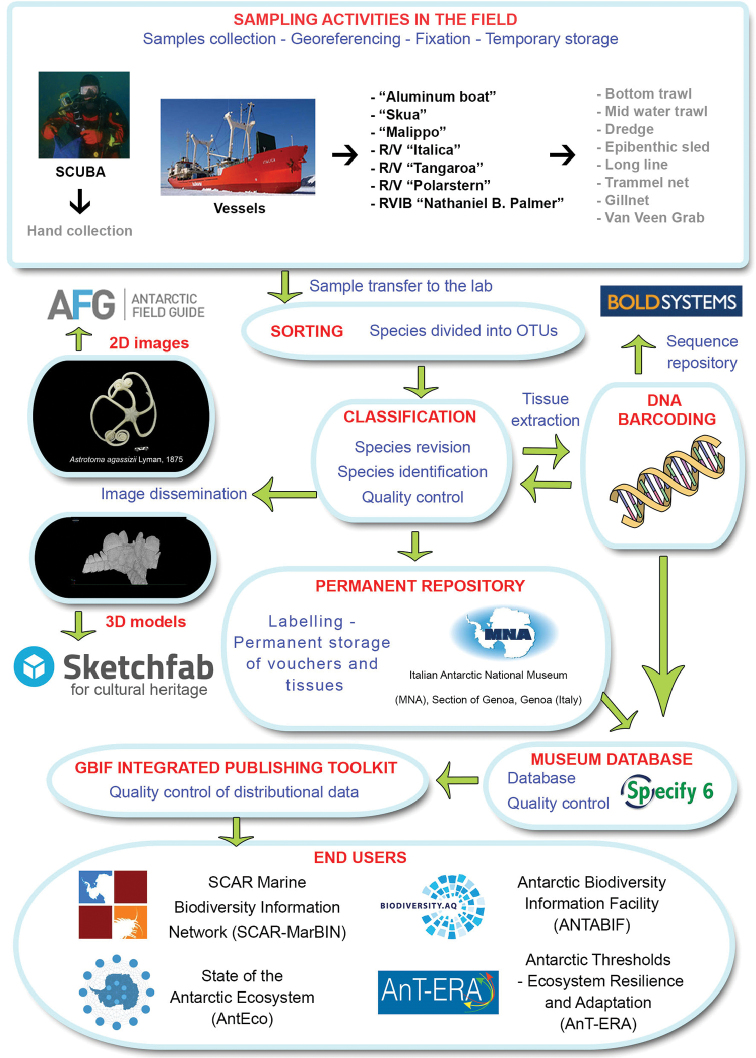
Flowchart depicting major steps in dataset development and publishing, from sample collection to the production of a virtual collection of the museum’s 3D vouchers.

**Method step description**: See sampling description below and flowchart of Figure 1.

**Figure 2.**
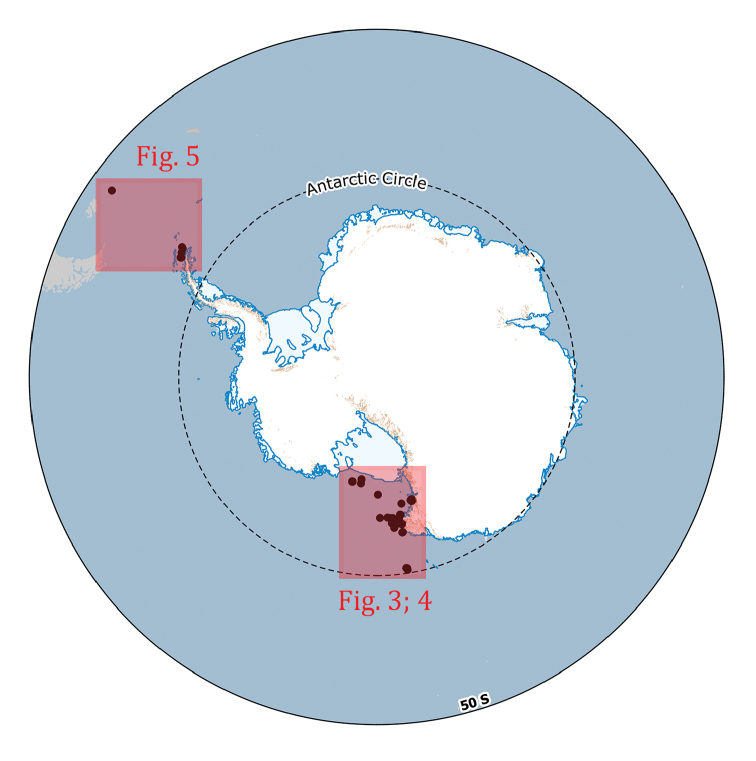
Overview map depicting the wideness of the area containing the sampling stations of the dataset. Areas in red are shown in detail in figures 3, 4 and 5.

**Figure 3.**
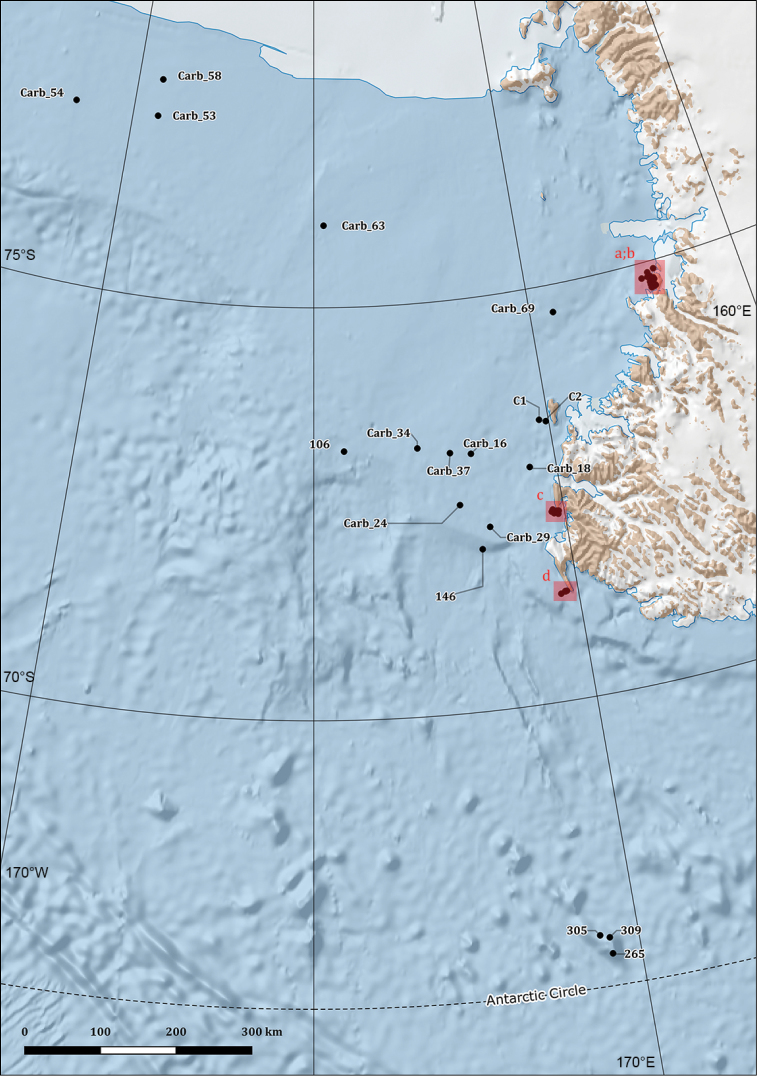
Map of the Ross Sea sampling locations.

**Figure 4.**
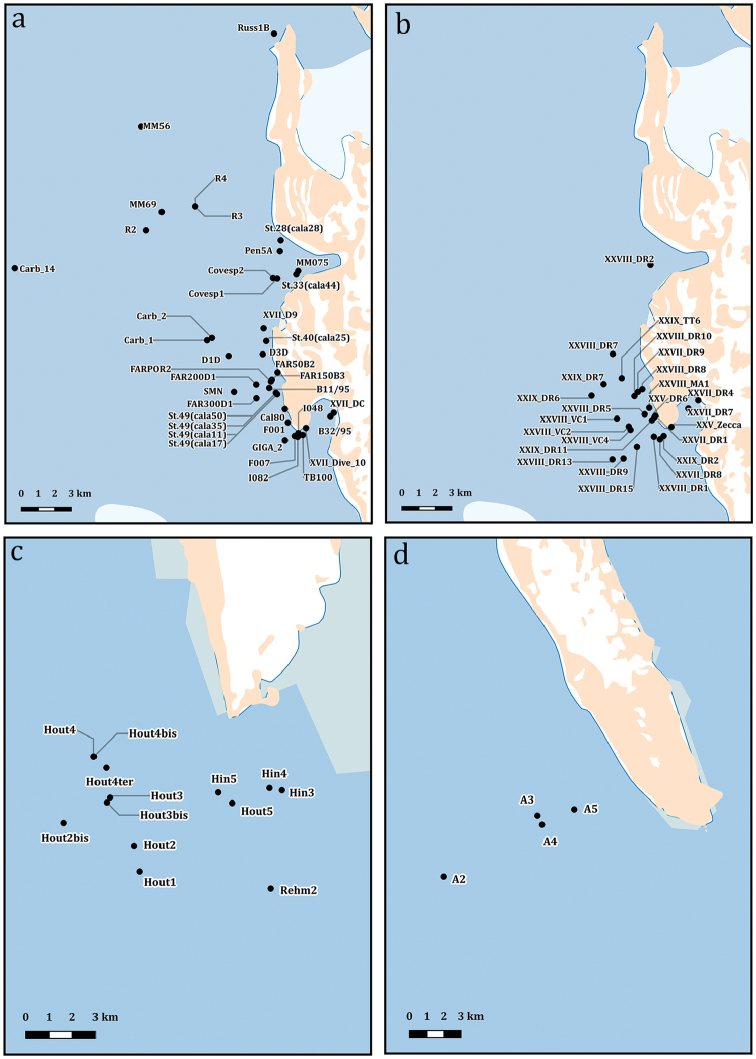
Highlight of the sampling sites in Terra Nova Bay (**a** sampling locations from 1988 to 2004 **b** sampling locations from 2009 to 2014), Cape Hallett (**c**) and Cape Adare (**d**) areas.

**Figure 5.**
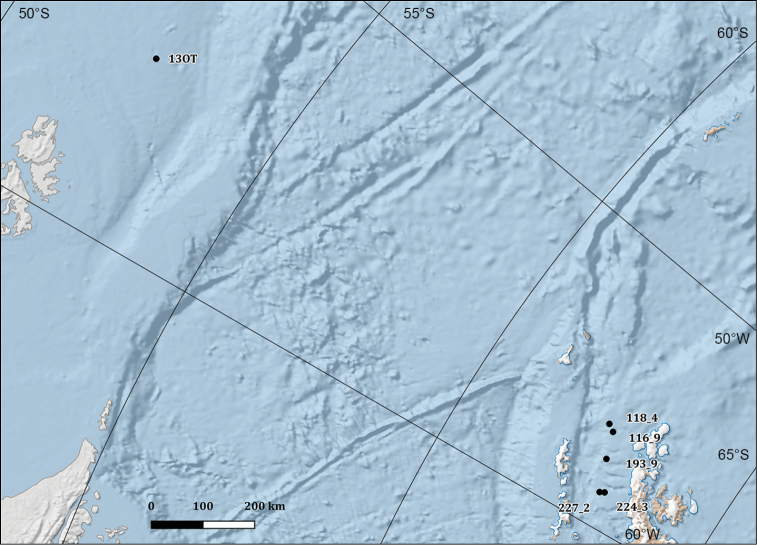
Map with the sampling sites in the Falkland Islands (Islas Malvinas) and Bransfield Strait

**Study extent description**: The Ophiuroidea distributional data here considered originated from 106 different sampling stations ranging between 21 and 1652 metres of depth (Figures 2, 3, 4, and 5) and investigated in the framework of different research expeditions from 1987 to 2014.

**Figure 6.**
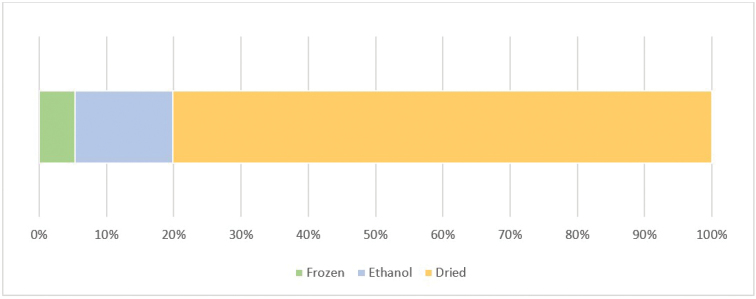
Number of individuals by preservation method stored at MNA. Dried specimens percentage in orange (~80%), ethanol in blue (~15%), and frozen in green (~5%)

Sampling description: The material was collected in the framework of several expeditions of the Italian National Antarctic Research Program (PNRA), of the National Institute of Water and Atmospheric Research (NIWA), of the Alfred Wegener Institute (AWI) and of the USA Antarctic Program. Sampling was performed through the deployment of a variety of sampling gears. Benthic sampling under the Italian PNRA was mainly performed by using a heterogeneous set of dredges (i.e. Charcot dredge and Triangular dredges) and Van Veen grabs of different volumes, plus an unconventional set of gears for sampling benthic fauna such as gillnets, trammel nets, long lines, also comprising a mid-water trawls (i.e. Multiple Net Tucker Trawl and Small Hamburg Plankton Net) that opportunistically collected benthic specimens due to accidental contact with the bottom during gear deployment failures. Two samples were collected during the “XVII” and “XXV” PNRA expeditions by SCUBA divers along the rocky cliffs of Tethys Bay. The NIWA expedition “TAN0802 IPY-CAML” (2008) used an epibenthic sled (“seamounts sled”; SEL) and a rough-bottom trawl (ORH). The German expedition “ANT-XXIX/3” (PS81) collected the material through the deployment of an Agassiz trawl while an Otter trawl was used to sample the single specimen collected during the ICEFISH 2004 cruise. After the material has been acquired by the MNA and included in the collections, all the specimens were classified to the lowest possible taxonomical resolution on a morphological base mainly by following Fell (1961). Several people contributed to the classification of samples on a morphological base: Matteo Cecchetto, Mariachiara Chiantore (Chiantore et al. 2006), Alice Guzzi, Francesca De Domenico (De Domenico 2006; De Domenico et al. 2006), Maria Paola Ferranti (Ferranti 2004) and Chiara Torre (Torre 2006). Morphological determinations have also been crosschecked with COI barcodes in the framework of the PNRA projects 2010/A1.10 “BAMBi” (Barcoding of Antarctic Marine Biodiversity) and PNRA 2013/AZ1.15 “ISOBIOTOX” (stable ISOtope and molecular markers for the reconstruction of Antarctic trophic webs under the sea-ice influence: evaluation of robustness to BIOdiversity loss and heavy metals bioaccumulation-neuroTOXicity). DNA extraction and sequencing of partial cytochrome c oxidase subunit 1 (CO1) was carried out at the Canadian Centre for DNA Barcoding (University of Guelph, Ontario, Canada) and sequences were uploaded on the BOLD platform (Barcode Of Life Data systems, http://www.boldsystems.org). The primers used for amplification were LCOech1aF1 and HCO2198 (Folmer et al. 1994). The present ophiuroid dataset has been formatted in order to fulfil the Darwin Core standard protocol required by the OBIS scheme (http://iobis.org/data/schema-and-meta-data) and according to the SCAR-MarBIN Data Toolkit (available at http://www.scarmarbin.be/documents/SM-FATv1.zip). The dataset was uploaded into AntOBIS (the Antarctic thematic node of OBIS). Vouchers stored at the Italian National Antarctic Museum (MNA), Section of Genoa, Genoa (Italy) are preserved, according to the different lots, in 75% ethanol, frozen (-20°C) or dried (Fig. 6). The dataflow illustrating sampling, storing procedures and data, metadata and images availability is reported in Fig. 1.

**Quality control description**: Specimens were classified at the lowest possible taxonomic level and only those that have been classified at least at the genus level were included in the present dataset. Morphological classifications indicated the presence of 18 species in the Terra Nova Bay area, a number confirmed also by the molecular data (COI barcodes) obtained by sequencing a total of 167 specimens representative of all the different morphospecies found here. The outcomes of the molecular study will be the subject of another publication and here we release only the COI sequences corresponding to the specimens that have been used to produce 3D models.

During all the phases of sorting, classification, and storage of samples at the Italian National Antarctic Museum, quality control and data cleaning have been undertaken at various steps in order to produce quality data and make consistent cross-references between the database and samples’ labels (Fig. 1). The MNA uses an SQL-based database (Specify 6) to manage its collections and link all the data (photos, sequences, etc.) to the physical samples. Geo-referencing on board of the different research vessels was based on the interpolation of GPS satellite receivers and a gyrocompass. Station coordinates and sampling events were recorded during sampling activities based on various GPS systems.

## Taxonomic coverage

**General taxonomic coverage description**: This dataset focused on the Class Ophiuroidea (Kingdom Animalia, Phylum Echinodermata) and includes a total of 1595 specimens belonging to 17 genera and 35 different species. In the Southern Ocean, the Class Ophiuroidea numbers 219 species (Martin Ledo and Lopez-Gonzalez 2014), thus representing one of the most important benthic groups of the Antarctic and sub-Antarctic regions. Fell (1961) made a biogeographic analysis of ophiuroid distribution in the Ross Sea and supplied a dichotomous key. The first complete faunistic inventory of ophiuroids occurring in the Terra Nova Bay area was published by Chiantore et al. (2006). Our material contains specimens belonging to three species that have not been previously sampled or recognized: one specimen of *Amphiura joubini* Koehler, 1912, one specimen of Amphiura (Amphiura) angularis Lyman, 1879, and four specimens of *Ophiura flexibilis* (Koehler, 1911). These records have thus to be added to the Terra Nova Bay checklist provided by Chiantore et al. (2006) and increase the check-list of Terra Nova Bay ophiuroid species from 15 to 18. The determination of these new records was also molecularly cross-checked (and confirmed) through COI barcodes. The outcomes of the barcoding effort on Terra Nova Bay ophiuroids and other echinoderm classes will be the subject of a separate contribution.

## Taxonomic ranks

**Kingdom**: Animalia

**Phylum**: Echinodermata

**Class**: Ophiuroidea

**Order**: Euryalida

**Family**: Gorgonocephalidae

**Genera**: *Astrotoma*, *Astrochlamys*, *Gorgonocephalus*

**Species**: *Astrochlamys bruneus*; *Astrochlamys sol*; *Astrotoma agassizii*; *Gorgonocephalus* sp.

**Kingdom**: Animalia

**Phylum**: Echinodermata

**Class**: Ophiuroidea

**Order**: Ophiurida

**Family**: Amphiuridae, Ophiacantidae, Ophiolepididae, Ophiuridae, Ophiuridae

**Genera**: *Amphiura*; *Ophiacantha*; *Ophiocamax*; *Ophioceres*; *Ophiocten*; *Ophiogona*; *Ophioleuce*; *Ophiolimna*; *Ophionotus*; *Ophioperla*; *Ophioplinthus*; *Ophiosparte*; *Ophiosteira*; *Ophiura*

**Species**: *Amphiura algida*; Amphiura (Amphiura) angularis; *Amphiura belgicae*; *Amphiura joubini*; *Amphiura* sp.; *Ophiacantha antarctica*; *Ophiacantha pentactis*; *Ophiacantha* sp.; *Ophiacantha vivipara*; *Ophiocamax gigas*; *Ophioceres incipiens*; *Ophiocten dubium*; *Ophiocten megaloplax*; *Ophiogona doederleini*; *Ophioleuce regulare*; *Ophiolimna antarctica*; *Ophionotus victoriae*; *Ophioperla koehleri*; *Ophioplinthus brevirima*; *Ophioplinthus gelida*; *Ophioplinthus martensi*; *Ophioplinthus* sp.; *Ophiosparte gigas*; *Ophiosteira antarctica*; *Ophiosteira bullivanti*; *Ophiosteira echinulata*; *Ophiura ambigua*; Ophiura (Ophiuroglypha) carinifera; *Ophiura crassa*; *Ophiura flexibilis*; *Ophiura* sp.

## Spatial coverage

**General geographic description**: The Ross Sea, the Bransfield Strait in the Antarctic Peninsula and southeast of the Falkland Islands (Islas Malvinas) (Figure 2, 3, 4 and 5).

## Coordinates

PNRA III expedition: -74.7125 and -74.79167; 164.03000 and 164.13667

PNRA V expedition: -74.68745 and -74.87100; 164.02433 and 164.26083

PNRA IX expedition: -74.72000; 164.14000

PNRA X expedition: -74.69320 and -74.71688; 164.04272 and 164.14700

PNRA XIII expedition: -74.68750; 164.14550

PNRA XIV expedition: -74.68762 and -74.89950; 163.93748 and 164.10728

PNRA XV expedition: -74.71343 and -74.72053; 164.17060 and 164.17783

PNRA XVII expedition: -72.20967 and -77.65133; 164.03418 and -166.79183

PNRA XIX expedition: -71.28833 and -74.82167; 164.19167 and 170.65333

PNRA XXV expedition: -74.69027 and -74.69768; 164.10255 and 164.13108

PNRA XXVII expedition: -74.68562 and -74.71337; 164.03502 and 164.14903

PNRA XXVIII expedition: -74.68090 and -74.77737; 164.05285 and 164.23640

PNRA XXIX expedition: -74.68677 and -74.72283; 164.12278 and 164.24206

New Zealand TAN0802 IPY-CAML voyage: -66.93250 and -73.24817; 170.87700 and 178.72367

German ANT-XXIX/3 (PS81) expedition: -62.43250 and -63.00883; -56.28767 and -58.68483

American ICEFISH 2004 cruise: -52.48498; -54.86978

## Temporal coverage

PNRA III expedition (1987/1988): January 15, 1988 - February 2, 1988

PNRA V expedition (1989/1990): December 25, 1989 - December 29, 1989

PNRA IX expedition (1993/1994): December 17, 1993

PNRA X expedition (1994/1995): January 25, 1995 - February 8, 1995

PNRA XIII expedition (1997/1998): February 21, 1998

PNRA XIV expedition (1998/1999): January 25, 1999 - February 10, 1999

PNRA XV expedition (1999/2000): February 10, 2000

PNRA XVII expedition (2001/2002): January 4, 2002 - February 10, 2002

PNRA XIX expedition (2003/2004): February 4, 2004 - February 21, 2004

PNRA XXV expedition (2009/2010): December 10, 2009 - January 11, 2010

PNRA XXVII expedition (2011/2012): January 27, 2012 - February 3, 2012

PNRA XXVIII expedition (2012/2013): January 9, 2013 - January 31, 2013

PNRA XXIX expedition (2013/2014): January 16, 2014 - February 3, 2014

New Zealand TAN0802 IPY-CAML voyage (2008): February 19, 2008 - March 14, 2008

German ANT-XXIX/3 (PS81) expedition (2013): January 26, 2013 - March 5, 2013

American ICEFISH 2004 cruise (2004): June 26, 1905

## Natural collections description

**Parent collection identifier**: Italian National Antarctic Museum (MNA) Section of Genoa, Italy

**Collection name**: MNA (Section of Genoa) Invertebrate Collection – Antarctic and sub-Antarctic Ophiuroidea

**Specimen preservation method**: Part of the material collected during the older expeditions (roughly from 1988 to 2004) was fixed in ethanol or formalin immediately after isolation and kept in tubes or vials for further studies, while the remaining samples were dried out to facilitate morphological identifications. The specimens corresponding to these older expeditions are therefore generally not usable for molecular analyses. Instead, the material collected during recent expeditions (i.e. roughly from 2005 to 2014) was fixed in 75% ethanol or frozen immediately after isolation and kept in the same condition in order to preserve the DNA quality and integrity (Fig. 6). Molecular data were obtained mainly from this second set of samples. All vouchers are now stored in the collections of the Italian National Antarctic Museum (MNA Section of Genoa, Italy).

**Figure 7.**
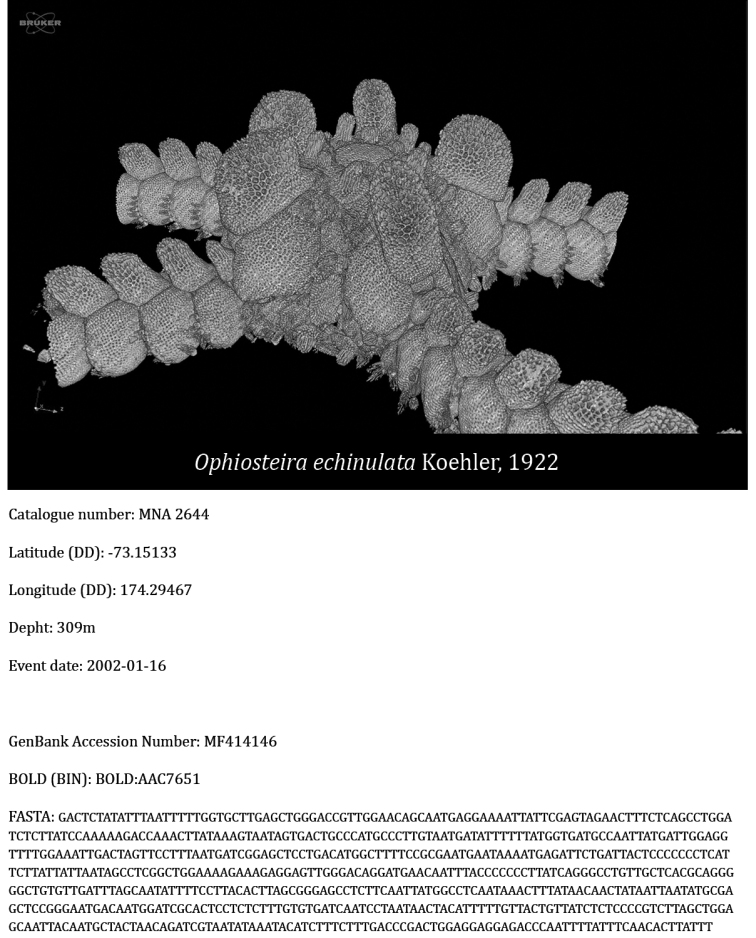
Video of the 3D model of *O. echinulata* (MNA 2644); Catalogue number, latitude (DD), longitude (DD), depth and event date as described in the section “Dataset description”, GenBank Accession number, Barcode Index Number from the Bold system and the complete COI sequence in FASTA format. Video available at: https://youtu.be/Sq6au-_CHy0

**Figure 8.**
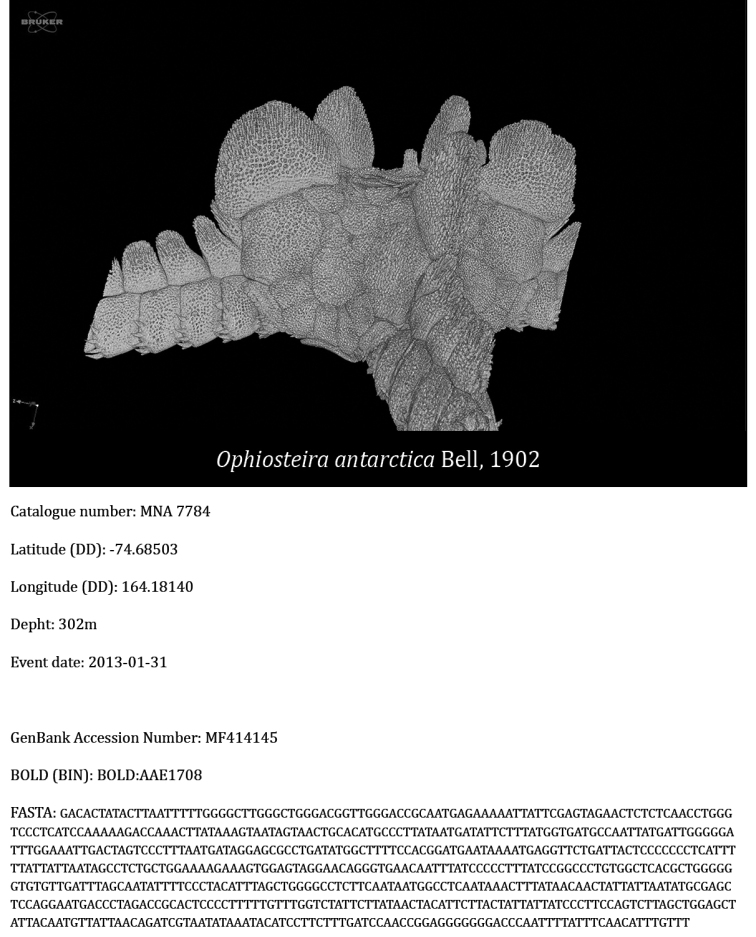
Video of the 3D model of *O. antarctica* (MNA 7784); Catalogue number, latitude (DD), longitude (DD), depth and event date as described in the section “Dataset description”; GenBank Accession number, the Barcode Index Number from the Bold system and the complete COI sequence in FASTA format. Video available at: https://youtu.be/Z72GryamWZY

**Figure 9.**
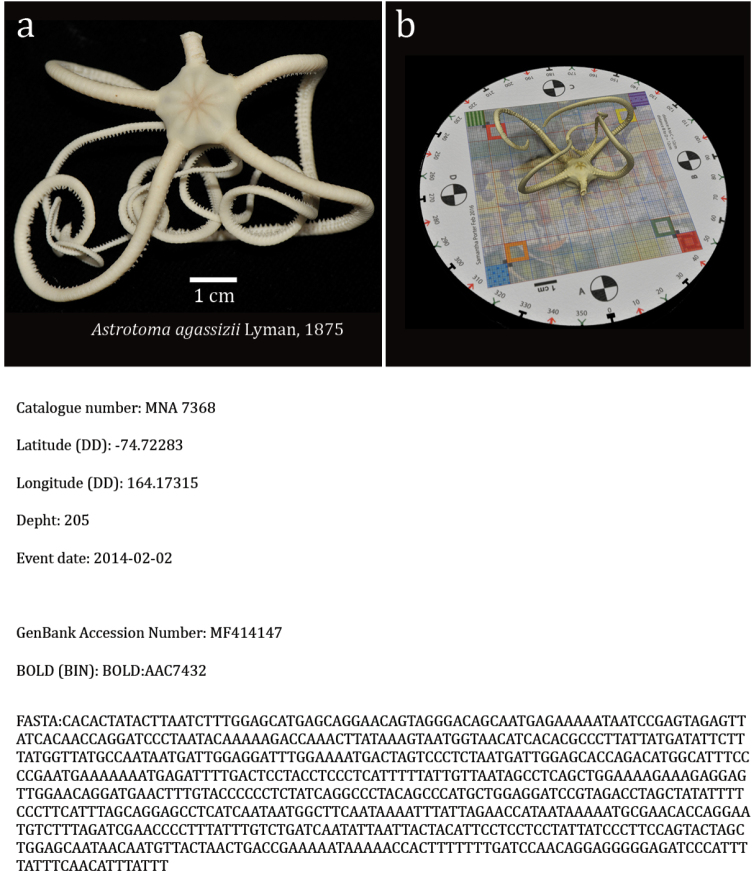
**a** The *A. agassizii* (MNA 7368) specimen used for photogrammetry documented immediately after collecting **b** screenshot of the 3D model based on photogrammetry at the mesh reconstruction stage (the marker showed as model background is available as supplementary material in Porter et al. 2016); Catalogue number, latitude (DD), longitude (DD), depth and event date as described in the section “Dataset description”; GenBank Accession number, the Barcode Index Number from the Bold system and the complete COI sequence in FASTA format.

**Virtual collection of vouchers and 3D models**: 3D models were obtained from three barcoded specimens of the species *Ophiosteira echinulata* (MNA 2644) (Fig. 7), *O. antarctica* (MNA 7784) (Fig. 8), and *Astrotoma agassizii* (MNA 7368) (Fig. 9). The first two models were obtained through micro-CT imaging performed at the Department of Geosciences (University of Padua) by using a bench-top Skyscan 1172 micro-CT system (Bruker), equipped with a Hamamatsu 100/250 microfocus X-ray source (80 kV, 124 μA) and a Hamamatsu C9300 11 megapixel camera (with a pixel size of 8.68 μm) filtered by a 0.5 mm Al foil. Projection images were acquired with a 1700 ms exposure time every 0.25° over 360° rotation, averaged over 10 frames and in vertical random movement mode to minimise noise. The run time for each scan was about 500 min. Samples were kept under ethanol in a polyester tube in order to avoid deterioration during acquisition. Cross-section slices were reconstructed from raw projection images using NRecon software program provided by Bruker, applying thermal correction, misalignment compensation, ring artefact reduction, and beam hardening correction. The exclusion of a binning procedure during acquisition and reconstruction allowed maximising spatial resolution obtaining 4K reconstructed images with voxel size of 4.94 μm (sample MNA 7784) and 3.95 μm (sample MNA 2644), respectively. Three-dimensional rendering and animations were performed with CTVox software package (Bruker).

The third model (MNA 7368) was produced by using photogrammetry and assembling pictures taken with a Nikon D700 equipped with a Nikon AF-S Micro Nikkor 105 mm (1: 2.8G ED) lens (corresponding to a resolution of 4256 x 2832 and a pixel size of 8.46 x 8.46 μm) in Agisoft Photoscan Professional 1.3.1. The MNA 3D models based on materials curated at the museum will be available from the MNA web site (www.mna.it) and from the Sketchfab gallery dedicated to cultural heritage and museums (https://sketchfab.com/MNA).

## Datasets

**Dataset description**: This dataset contains data about the Phylum Echinodermata, Class Ophiuroidea, from the Antarctic and sub-Antarctic regions. Combined, it includes 35 different species corresponding to a total of 1595 specimens. The validity and synonyms of each species name were checked in WORMS (World Register of Marine Species; http://www.marinespecies.org; last check has been made in 2017-04-11). The Darwin Core elements included in the dataset are: occurrence ID (a global unique identifier in the form “urn:catalog:[institutionCode]:[collectionCode]:[catalogNumber]”), institution code (the name of the institution where the samples are kept, i.e. MNA), basis of record (all records consist in preserved specimens), preparations (i.e. the preservation method), catalogue number (i.e. MNA catalogue number), individual count, sampling protocol (i.e. sampling gear), event date (i.e. year, month and day), event remarks (i.e. expedition), field number (i.e. sampling station), field notes (field number map acronym, i.e. name of the sampling stations as showed on maps), maximum depth in meters, decimal latitude (DD), decimal longitude (DD) and scientific name.

**Object name**: MNA (Section of Genoa) – Antarctic and sub-Antarctic Ophiuroidea

**Character encoding**: UTF-8

**Format name**: Darwin Core Archive format

**Format version**: 1.0

**Distribution**: http://ipt.biodiversity.aq/resource.do?r=mna_antarctic_ophiuroidea

**Language**: English

**Metadata language**: English

**License of use**: This dataset [MNA (Section of Genoa) – Antarctic and sub-Antarctic Ophiuroidea] is made available under the Creative Commons Attribution License (CC-BY) 4.0: http://www.creativecommons.org/licenses/by/4.0/legalcode

**Date of metadata creation**: 2017-05-04

**Hierarchy level**: Dataset

## References cited within the metadata

Chiantore M, Guidetti M, Cavallero M, De Domenico F, Albertelli G, Cattaneo-Vietti R (2006) Sea urchins, sea stars and brittle stars from Terra Nova Bay (Ross Sea, Antarctica). Polar Biology 29(6): 467. https://doi.org/10.1007/s00300-005-0077-2

De Domenico F (2004) Biodiversità ed ecologia degli Echinodermi della Terra Vittoria (Mare di Ross). PhD thesis, Genoa, Italy: University of Genoa.

De Domenico F, Chiantore M, Buongiovanni S, Ferranti MP, Ghione S, Thrush S, Cummings V, Hewitt J, Kroeger K, Cattaneo-Vietti R (2006) Latitude versus local effects on echinoderm assemblages along the Victoria Land coast, Ross Sea, Antarctica. Antarctic Science 18(04): 655–662. https://doi.org/10.1017/S095410200600068X

Ferranti MP (2004) Distribuzione batimetrica e latitudinale degli ofiuroidei nel settore occidentale del Mare di Ross (Antartide). Bachelor thesis, University of Genoa, Genoa, Italy.

Torre C (2004) Variabilità fenotipica di alcuni ofiuroidi (Echinodermata, Ophiuroidea) lungo la Terra Vittoria (Mare di Ross, Antartide). Bachelor thesis, University of Genoa, Genoa, Italy.

## Publications based on this dataset

Chiantore M, Guidetti M, Cavallero M, De Domenico F, Albertelli G, Cattaneo-Vietti R (2006) Sea urchins, sea stars and brittle stars from Terra Nova Bay (Ross Sea, Antarctica). Polar Biology 29(6): 467. https://doi.org/10.1007/s00300-005-0077-2

De Domenico F, Chiantore M, Buongiovanni S, Ferranti MP, Ghione S, Thrush S, Cummings V, Hewitt J, Kroeger K, Cattaneo-Vietti R (2006) Latitude versus local effects on echinoderm assemblages along the Victoria Land coast, Ross Sea, Antarctica. Antarctic Science 18(04): 655–662. https://doi.org/10.1017/S095410200600068X
